# Kinetics of Immune Subsets in COVID-19 Patients Treated with Corticosteroids

**DOI:** 10.3390/v15010051

**Published:** 2022-12-24

**Authors:** Apostolos Georgios Pappas, Anna-Louiza Chaliasou, Andreas Panagopoulos, Konstantina Dede, Stavroula Daskalopoulou, Evie Moniem, Eftychia Polydora, Eirini Grigoriou, Katherina Psarra, Alexandra Tsirogianni, Ioannis Kalomenidis

**Affiliations:** 1 1st Department of Critical Care and Pulmonary Medicine, School of Medicine, National and Kapodistrian University of Athens, “Evangelismos” General Hospital, 10676 Athens, Greece; 2 Intensive Care Unit, “G. Gennimatas” General Hospital, 11527 Athens, Greece; 3 COVID-19 Unit, “Evangelismos” General Hospital, 10676 Athens, Greece; 4 4th Department of Internal Medicine, “Evangelismos” General Hospital, 10676 Athens, Greece; 5 3rd Department of Internal Medicine, “Evangelismos” General Hospital, 10676 Athens, Greece; 6 5th Department of Internal Medicine, “Evangelismos” General Hospital, 10676 Athens, Greece; 7 Department of Immunology—Histocompatibility, “Evangelismos” General Hospital, 10676 Athens, Greece

**Keywords:** COVID-19, SARS-CoV-2, lymphopenia, dexamethasone, corticosteroids, lymphocyte kinetics

## Abstract

Rationale: Changes in anti-SARS-CoV-2 defense immune subsets in patients treated with dexamethasone (DXM) for severe COVID-19 and their relation to disease outcomes are poorly understood. Methods: Blood-lymphocyte subsets of 110 hospitalized COVID-19 patients were prospectively examined. A first sample was taken at enrollment and a second one 7–10 days later. Total B-, T-lymphocytes, CD4+, CD8+, T-regulatory (Treg), Natural-Killer (NK) and NK T-cells were counted using flow cytometry. Results: At enrollment, patients with respiratory failure, characterized by DXM failure (intubation/death) or DXM success (hospital discharge) exhibited significantly fewer CD3+, CD4+ and CD8+ cells and B-lymphocytes compared to the control group (no respiratory failure/no DXM). At the time of treatment completion, the DXM-failure group exhibited significantly fewer CD3+, CD4+ and CD8+ cells, memory CD4+ and CD8+ T-lymphocytes, compared to the control and the DXM-success groups and fewer activated CD4+ T-lymphocytes, Tregs and NK cells compared to the control group. At the time of treatment completion, the number of all investigated lymphocyte subsets increased in the DXM-success group and was similar to those of the control group. NK cells significantly decreased over time in the DXM-failure group. Conclusion: The lymphocyte kinetics differ between DXM-treated and control COVID-19 patients and are associated with clinical outcomes.

## 1. Introduction

COVID-19, the disease caused by the novel SARS-CoV-2 [1], is still affecting millions of people worldwide [2]. Despite the development of safe and effective vaccines [3], the continuous evolution of the virus causes newly-confirmed cases and re-infections [2], fueling surges that impose pressure on healthcare systems.

COVID-19 presents a highly variable clinical course, ranging from asymptomatic infection to critical disease, with the development of acute respiratory distress syndrome (ARDS) [4]. Advanced disease is characterized by a hyper-inflammatory state, with high levels of circulating pro-inflammatory cytokines, such as Interleukin-6 (IL-6), Tumor Necrosis Factor-alpha (TNF-α) and interferon-gamma (IFN-γ) [5], which have been blamed for the extensive COVID-19-related tissue damage [6]. Not surprisingly then, immunomodulatory and anti-inflammatory agents have been demonstrated to improve outcomes in patients with severe disease [7,8]. Among them, dexamethasone was the first to show a survival benefit in these patients [9]. However, the immune response to SARS-CoV-2 is largely variable [5,10,11], with dysfunctional interferon signaling [5], natural-killer (NK) cell exhaustion [12], low number of naïve T- and B-lymphocytes [10,13] and the absence of SARS-CoV-2-specific memory T-cells [14,15] being associated with dismal outcomes [5,10]. This variability may be linked to the observation that corticosteroids could be more beneficial in certain groups of patients than others [16,17,18,19]. Furthermore, temporal alterations of critical to anti-SARS-CoV2 defense immune subsets in hospitalized COVID-19 patients and their relation to the clinical outcomes are poorly defined.

The aim of this study was to evaluate the lymphocyte sub-populations in hospitalized patients with COVID-19, with or without corticosteroid treatment and to investigate the link between these changes and outcomes. We hypothesized that the baseline profile of lymphocyte subsets would differ between patients with and those without respiratory failure. We also hypothesized that, among patients with respiratory failure, the baseline profile and the changes in lymphocyte subsets during steroid treatment would differ between those who deteriorated and those who responded to treatment.

## 2. Materials and Methods

### 2.1. Patients and Data Collection

We prospectively enrolled patients admitted to “Evangelismos” General Hospital (Athens, Greece) between 29 September 2020 and 24 June 2021. Inclusion criteria were: (1) positive nasopharyngeal swab Polymerase Chain Reaction (PCR) test for SARS-CoV-2 (the dominant SARS-CoV-2 variant during the study period was alpha variant (B.1.1.7)) and (2) time from admission less than 3 days. Exclusion criteria were the following: (1) age under 18 years old, (2) already receiving corticosteroids, (3) contraindication to receive corticosteroids, (4) being a candidate to receive other immunomodulatory drugs on top of steroids, as judged by the clinical team, (5) previous treatment with B-cell depleting agents, (6) hematologic malignancies, (7) pregnancy and (8) participation in a clinical trial. Patients were followed until discharge or hospital death. The study was conducted according to the principles of the Declaration of Helsinki and was approved by the Ethics Committee of “Evangelismos” General Hospital (Athens, Greece), protocol number: 596/17.12.2020 and by the Ethics Committee of Medical School of the National and Kapodistrian University of Athens (Athens, Greece), protocol number: 449/14.01.2021. All participants signed an informed consent form.

### 2.2. Treatment and Outcomes

The patients were treated according to the Greek National Public Health Organization guidelines for COVID-19 (eody.gov.gr/en/covid-19/ (accessed on 27 August 2020)). During the study period, patients with COVID-19 pneumonia, without respiratory failure, received remdesivir, 200 mg intravenously for one day and then 100 mg for another 4 to 9 days. Those with COVID-19 pneumonia and respiratory failure were also treated with intravenous dexamethasone, 6 mg per day (or with an equivalent dose of another steroid) for 7 or 10 days. All patients received supportive care, including a prophylactic dose of low molecular weight heparin (LMWH) and antibiotic therapy, depending on the judgment of the supervising physician. Oxygen therapy was delivered by nasal cannula, venturi mask, non-rebreather mask and high-flow nasal oxygen therapy (HFNO), if available. In the case of HFNO failure, patients were intubated and received mechanical ventilation. For the purpose of the study, intubation or death was considered “treatment failure”, while hospital discharge without the need for intubation and mechanical ventilation was considered “treatment success”.

### 2.3. Sampling Schedule

Once patients signed the consent form, a blood sample was taken for lymphocyte subtype evaluation. In patients with the first sample taken when not in oxygen therapy, a second blood sample was taken in the case they progressed to respiratory failure before the administration of corticosteroids. A final blood sample was taken 10 days (or the closest working day to day 10 if that was at the weekend or a holiday) after the first sample from patients that did not receive corticosteroids or 10 days after the second sample from the patients who developed respiratory failure and received corticosteroids. The final blood sample could be taken earlier if the patient improved and was about to be discharged from the hospital or if they were moribund. Those recruited while being in respiratory failure had two samples taken: one before corticosteroid commencement and the second one 10 days after the first, or earlier as described above.

### 2.4. Assessment of Lymphocyte Subpopulations

The following immune subsets were evaluated using flow cytometry: CD3+ T- lymphocytes, CD3+CD4+ T-helper lymphocytes, CD3+CD8+ T-cytotoxic lymphocytes, CD19+ B-lymphocytes, CD3-CD16/56+ Natural Killer (NK) cells, CD3+CD16/56+ Natural killer-like T-lymphocytes (NKT cells), CD3+CD4+CD25strCD127- Regulatory T-cells (Tregs), CD3+CD4+CD45RA+ and CD3+CD8+CD45RA+ (Naϊve) CD4+ and CD8+ T-lymphocytes, CD3+CD4+CD45RO+ and CD3+CD8+CD45RO+, memory CD4+ and CD8+ lymphocytes. HLADR+CD4+ and HLADR+CD8+ lymphocytes and CD38+CD4+ and CD38+CD8+ lymphocytes represent activated CD4 and CD8 T-cells, respectively. Immunophenotyping was carried out at the Department of Immunology-Histocompatibility of “Evangelismos” General Hospital (Athens, Greece). The analysis was performed on Navios and Navios EX Flow Cytometer with the Navios software (Beckman Coulter, Brea, CA, USA). The following antibody panels were used to stain the blood samples that were collected in ethylenediaminetetraacetic acid (EDTA) tubes: CD45-FITC, CD4-FITC, CD45RA-FITC, CD3-PC7, CD4-PE, CD8-PC5.5, CD38-APC and CD45-BV 570 (Biolegend, San Diego, CA, USA), CD19-PC5, CD127-PE, CD25-PC5, CD45RO-PE, CD4-APC-750, HLADR-PB (Beckman Coulter, Brea, CA, USA) and CD16/56-PE (Cytognos, Salamanca, Spain). For each sample, we used four fluorochrome combinations in four tubes that were the following: (i) CD45-FITC/CD4-PE/CD8-PC5.5/CD3-PC7, (ii) CD45-FITC/CD16/56-PE/CD19-PC5/CD3-PC7, (iii) CD45RA-FITC/CD45RO-PE/CD8-PC5.5/CD3-PC7/CD38-APC/CD4-APC-750/HLADR-PB/CD45-BV 570, (iv) CD4-FITC/CD127-PE/CD25-PC5/CD3-PC7.

### 2.5. Statistical Analysis

Continuous variables are presented as mean ± standard error of the mean (SEM), if they were normally distributed and median (25th–75th interquartile range-IQR) if they were not. The comparison between lymphocyte-subset counts at the same time point was performed using the one-way analysis of variance (ANOVA) with Bonferroni post-hoc or the Kruskal–Wallis with Dunn post-hoc tests, depending on whether the distributions were Gaussian or not. The comparison between patients’ features and lymphocyte populations between different time points was performed using Student’s *T*-test or the Mann–Whitney U-test, depending on whether the distributions were normal or not. Categorical data were assessed using the chi-square test. A *p*-value < 0.05 was considered statistically significant. Calculations were performed using Graphpad Prism Software (San Diego, CA, US).

## 3. Results

### 3.1. Patients and Samples

A total of 110 patients were enrolled in the study. A total of 66 patients suffered from respiratory failure at the time of recruitment, while 42 did not present respiratory failure at any time and constituted the control group. These two groups had two samples taken. Two patients were enrolled while having pneumonia with no respiratory failure, who deteriorated within 24 h and required supplemental oxygen. They had a second sample taken before the initiation of dexamethasone and a third, as described in the methods section. We decided to add those 2 patients to the broader group of 66 patients who were enrolled while on respiratory failure. For this reason, the first sample of those two patients was ignored, and the sample that was obtained when they developed respiratory failure was considered that corresponding to the enrollment. All patients without respiratory failure were discharged from the hospital. Among the 68 patients who developed respiratory failure and were treated with corticosteroids, 56 were discharged from the hospital without being intubated and mechanically ventilated, while 11 patients required mechanical ventilation due to severe ARDS and 7 of them eventually died. Another patient died due to respiratory failure without being intubated because oxygen therapy was set as the ceiling treatment.

Therefore, we analyzed three patient groups: (1) those without respiratory failure or dexamethasone treatment (control group), (2) those with respiratory failure successfully treated with dexamethasone (DXM-success) and (3) those with respiratory failure, who received dexamethasone, but they were intubated or died (DXM-failure). We analyzed the immune subsets using samples from the first time point (time point A (tpA)) and the second time point (time point B (tpB)), as previously explained. The characteristics of the patients at the time of enrollment are displayed in Table 1, and their laboratory findings are shown in Table S1. The patients of the control group were younger, had fewer comorbidities (as it is indicated by the lower Charlson Comorbidity Index—CCI score) and they were presented with milder disease (as it is indicated by the lower NEWS2 score, the lower respiratory rate and the less extended chest X-ray infiltrates) compared to the DXM-treated groups. On the other hand, the demographic characteristics of the patients in the DXM-success and DXM-failure groups were similar.

The timing of the acquisition of the samples is displayed in Table S2. The time distance between symptom onset or admission and tpA did not differ among the three groups. TpB occurred earlier in patients discharged without requiring dexamethasone treatment than in patients of either of the two dexamethasone groups. The time distance between symptom onset or admission and tpB tended to be longer in those with treatment failure than in those with treatment success, but the difference was not statistically significant.

### 3.2. Kinetics of CD4 and CD8 Cells

**Figure 1:**
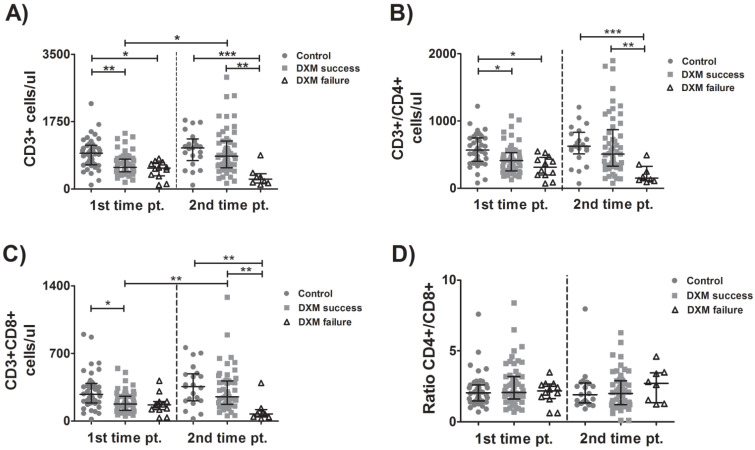
Kinetics of CD4 and CD8 T-lymphocytes. Lymphocyte subpopulations were evaluated over time in blood samples from patients with COVID-19 who received (or not) treatment with dexamethasone. Total CD3+ cells (**A**), CD4+ T-lymphocytes (**B**), CD8+ T-lymphcytes (**C**) and ratio CD4+/CD8+ cells (**D**) were determined with flow cytometry. Data are presented as median (interquartile range—IQR). Multiple comparisons were made using Kruskal–Wallis with Dunn’s post-hoc tests. Comparisons between the two groups were made with Student’s *T*-test or the Mann–Whitney U-test as indicated. DXM: Dexamethasone. *p*-values < 0.05 were considered significant. * *p* < 0.05, ** *p* < 0.01, *** *p* < 0.001.

At patient enrollment (tpA), the total number of lymphocytes was higher in the control group than in either the DXM-success or DXM-failure groups. At tpB, both the control group and the DXM-success group had higher CD3+ counts than the DXM-failure group. These observations reflected the findings of CD3+ kinetics: while their number remained stable in the control group between tpA and tpB, those successfully treated with dexamethasone had increased lymphocyte counts over time. Patients with treatment failure displayed a non-significant fall in the CD3+ cells between enrollment and the event indicating treatment failure (Figure 1A). Similarly, CD4+ T-lymphocyte count was higher in the control group than either of the DXM group at enrollment and higher in the control group or DXM-success group than in the DXM-failure group at the time of treatment completion. However, significantly different changes over time were not observed in any of the three groups of patients (Figure 1B). CD8+ T-lymphocyte count was higher in the control group than in the DXM-success group at tpA and in the control or DXM-success group than in the DXM-failure group at tpB. The number of the CD8+ T-cells between tpA and tpB increased significantly in the DXM-success group (Figure 1C). No significant differences were observed concerning the CD4+/CD8+ ratio between groups or time points (Figure 1D). It should be noted, however, that patients with treatment failure displayed a fall in CD3+, CD4+ and CD8+ cells between enrollment and treatment completion, but these differences were not statistically significant, possibly due to the small number of observations in this specific group of patients.

### 3.3. Kinetics of Naïve and Memory Lymphocytes

**Figure 2:**
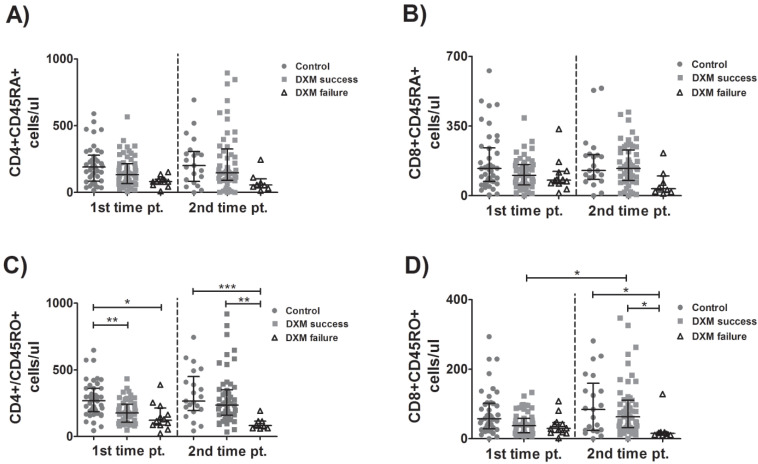
Kinetics of naïve and memory lymphocytes. Lymphocyte subpopulations were evaluated over time in blood samples from patients with COVID-19 who received (or not) treatment with dexamethasone. Naïve CD4+ (**A**), CD8+ (**B**) T-lymphocytes and memory CD4+ (**C**) and CD8+ (**D**) T-lymphocytes were determined with flow cytometry. Multiple comparisons were made using Kruskal–Wallis with Dunn’s post-hoc tests. Comparisons between the two groups were made with Student’s *T*-test or the Mann–Whitney U-test as indicated. DXM: Dexamethasone. *p*-values < 0.05 were considered significant. * *p* < 0.05, ** *p* < 0.01, *** *p* < 0.001.

As for CD4+ and CD8+ naïve lymphocytes, no significant differences were observed either between groups or between different time points (Figure 2A,B). In agreement with the patterns observed in total and CD4+ T-lymphocytes, memory CD4+ cell count was higher in the control group than in the DXM-success group and the DXM-failure group at tpA and higher in the control group or DXM-success group compared to the DXM-failure group at tpB (Figure 2C). The memory CD8+ T-lymphocyte count was higher in the control group or DXM-success group than in the DXM-failure group, and a significant increase in these cells was observed between time points in the DXM-success group (Figure 2D). Overall, patients with failure of DXM treatment tended to have lower numbers of naïve and memory lymphocytes and a downward trend of them over time as well. However, the differences were not statistically significant.

### 3.4. Kinetics of Activated Lymphocytes

**Figure 3:**
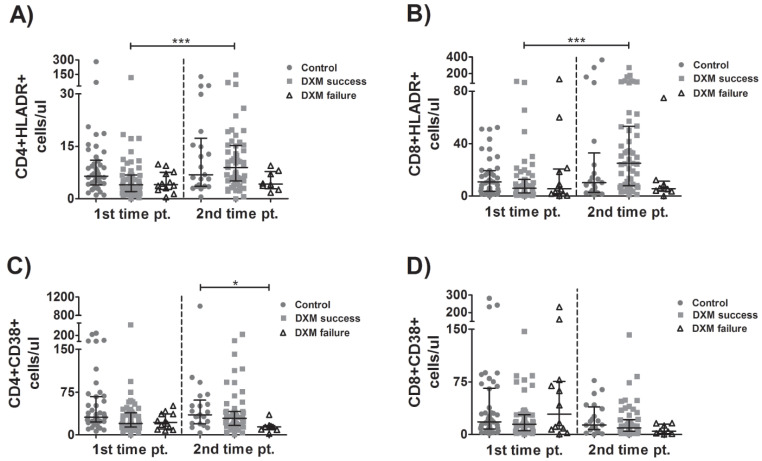
Kinetics of activated T-lymphocytes. Lymphocyte sub-populations were evaluated over time in blood samples from patients with COVID-19 who received (or not) treatment with dexamethasone. Activated CD4+ (**A**,**C**) T-lymphocytes and activated CD8+ (**B**,**D**) T-lymphocytes were determined with flow cytometry. Multiple comparisons were made using Kruskal–Wallis with Dunn’s post-hoc tests. Comparisons between the two groups were made with Student’s *T*-test or the Mann–Whitney U-test as indicated. DXM: Dexamethasone. *p*-values < 0.05 were considered significant. * *p* < 0.05, *** *p* < 0.001.

A significant increase in HLADR+CD4+ (Figure 3A) and HLADR+CD8+ cells (Figure 3B) between time points was observed in the patients successfully treated with corticosteroids. CD4+CD38+ cell counts were higher in the control than in DXM-failure group (Figure 3C). No other statistically significant changes in the activated lymphocytes either between groups or over time were observed (Figure 3A–D).

### 3.5. Kinetics of B-Cells, Tregs, NK and NKT Cells

**Figure 4:**
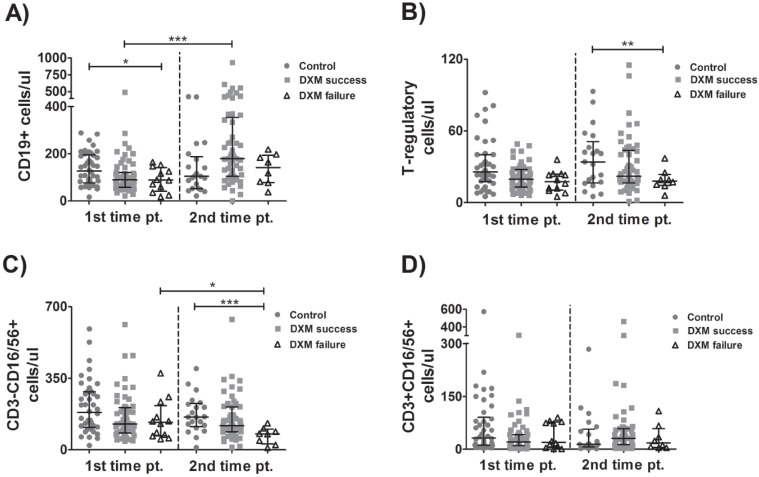
Kinetics of B-cells, Tregs, NK and NKT cells.Lymphocyte subpopulations were evaluated over time in blood samples from patients with COVID-19 who received (or not) treatment with dexamethasone. B-lymphocytes (**A**), T-regulatory cells-Tregs (**B**), Natural-killer cells (**C**) and Natural-killer T-cells (**D**) were determined with flow cytometry. Multiple comparisons were made using Kruskal–Wallis with Dunn’s post-hoc tests. Comparisons between the two groups were made with Student’s *T*-test or the Mann–Whitney U-test as indicated. DXM: Dexamethasone. *p*-values < 0.05 were considered significant. * *p* < 0.05, ** *p* < 0.01, *** *p* < 0.001.

At the time of enrollment, the B-cell count was higher in the control group than in the DXM-failure group and between tpA and tpB in both dexamethasone-treated groups. However, the increase was more pronounced and statistically significant in DXM-success patients (Figure 4A). Treg count was higher in the no-DXM group compared to the DXM-failure group (Figure 4B). NK cell count was higher in the control group than in the DXM-failure group at tpB and decreased significantly between time points in the DXM-failure group (Figure 4C). No significant difference in NKT-cell counts was observed between groups or between time points (Figure 4D).

## 4. Discussion

Here, we prospectively evaluated lymphocyte sub-populations in hospitalized patients with COVID-19 who presented respiratory failure and received treatment with dexamethasone. Patients without respiratory failure who were not treated with corticosteroids constituted the control group. We examined whether baseline profiles and changes in blood lymphocyte-subset counts over time were linked to treatment failure (DXM-failure), defined as intubation or death, or success (DXM-success), defined as hospital discharge without the need for intubation. Our main findings were: (1) At the time of enrollment, the DXM-failure group exhibited significantly fewer CD3+ cells, CD4+ T-lymphocytes, memory CD4+ T-lymphocytes and B-lymphocytes compared to the control group and similar lymphocyte subpopulation profiles to the DXM-success group. (2) At the time of the treatment completion, the DXM-failure group exhibited significantly fewer CD3+ cells, CD4+ and CD8+ T-lymphocytes, and memory CD4+ and CD8+ T-lymphocytes compared to the control and the DXM-success group and fewer activated CD4+ T-lymphocytes, Tregs and NK cells compared to the control group. (3) At the time of enrolment, DXM-success patients had fewer CD3+, CD4+, CD8+ and memory CD4+ cells compared to the control ones, while at the time of treatment completion, all investigated lymphocyte subsets did not differ between these two groups. (4) The number of all of the investigated lymphocyte subsets remained stable over time in the control group. NK cells significantly decreased over time in the DXM-failure group. On the other hand, the number of total CD3+ cells, CD8+ T-lymphocytes, memory and activated CD8+ T-lymphocytes, activated CD4+ T-lymphocytes and B-lymphocytes significantly increased overtime in the DXM-success group.

Lymphopenia is a hallmark of severe and critical COVID-19 [20,21,22,23] and is a predictor of upcoming respiratory failure in patients with SARS-CoV-2-related pneumonia [24]. In line with these observations, we here demonstrated that patients with severe COVID-19 had significantly fewer T- and B-lymphocytes compared to those with pneumonia and no respiratory failure and that the profile of lymphocyte subtypes was similar between patients who recovered after the administration of dexamethasone and those who progressed to intubation and/or death. Therefore, it appears that the baseline profile of the lymphocyte subsets investigated here could not predict the outcome in patients treated with corticosteroids for COVID-19-related respiratory failure. On the other hand, we demonstrated that in the patients successfully treated with corticosteroids (DXM-success group), the number of different lymphocyte sub-populations increased during treatment in a way that at the end of the dexamethasone delivery, the initial differences between the DXM-success and the control groups disappeared. This “normalization” of the lymphocyte profile comes in sharp contrast with the well-established [25,26] lymphocyte-depleting effects of dexamethasone, and it is in agreement with reports from the early phase of the pandemic, where all T-lymphocyte subsets rose to the normal level in convalescent patients who received no specific treatment for SARS-CoV-2 infection [27]. However, lymphocyte subset “normalization” was not observed in the patients who progressed to intubation and/or death, despite dexamethasone treatment. Overall, these findings support the notion that a dysregulated and ineffective immune response might underlie severe COVID-19 [5,10].

T-lymphocytes play a fundamental role in the limitation of SARS-CoV-2 infection [10]. Rapid induction of virus-specific CD4+ cells has been associated with a milder COVID-19 course [28], and their absence was linked to fatal outcomes in patients with COVID-19 [14]. Similarly, the presence of virus-specific CD8+ T-lymphocytes has been strongly associated with better outcomes in patients with SARS-CoV-2 infection [13,29]. Furthermore, a significant increase in the number of CD4+, CD8+ and activated T-lymphocytes was observed in patients who responded to dexamethasone (alone or in combination with interferon beta 1a) [30]. Similarly, in another group of patients who received different immunomodulatory agents (corticosteroids, immunoglobulin and interferon) alone or in combination, the low post-treatment number of CD8+ T-lymphocytes was linked to treatment failure [31]. These findings come alongside our observations that the patients without respiratory failure had more baseline CD4+ and CD8+ T-lymphocytes (compared to those with severe COVID-19) and a pronounced increase in activated and memory CD4+ T-cells and total and activated CD8+ T-cells over time was observed in the patients treated with dexamethasone who reached convalescence, but not in those who failed to recover.

NK cells are the innate immune subset with critical importance regarding the antiviral immune response [32,33]. Previous reports indicated that patients with severe COVID-19 exhibit fewer blood-circulating NK cells, which are characterized by a functionally exhausted phenotype [12]. In the present study, while the baseline NK counts did not differ between groups and did not predict the final outcome in the patients with respiratory failure, a striking fall of NK cells during dexamethasone treatment was observed in those who were intubated or died. On the contrary, in agreement with previous observations [34], we have shown that NK T-cell populations were not associated with COVID-19 severity and progression.

Along with innate and T-cell immunity, functional B-cells facilitate viral clearance through the production of SARS-CoV-2 neutralizing antibodies [10]. Patients with hematologic malignancies or patients receiving B-cell depletion therapy are characterized by the inability to effectively eliminate SARS-CoV-2 and are susceptible to a protracted disease course [35,36]. In our study, CD19+ cell numbers at baseline were lower in the DXM-failure compared to the control group and although their number was higher over time in all the patients who received dexamethasone, this increase was significant only in the patients with the optimal outcome. This finding may be explained by the fact that the majority of patients with SARS-CoV-2 infection seroconvert between 5–15 days post symptom onset [10,37], and the activation of naïve B-cells is required for the development of neutralizing antibodies [10,38]. On the contrary, other investigators showed that only patients with severe/critical COVID-19 exhibited an increase in the number of B-lymphocytes [39]. These divergent observations may be partly explained by differences in the treatment regimen; for example, in the study by Scalia et al., patients received corticosteroids before hospital admission, and some of them were also treated with tocilizumab during hospital stay [39].

The limitations of the present study include the following; (1) the small number of participants, especially in the DXM-failure group, may have hidden significant differences concerning observations in these patients; (2) the fact that the investigation of peripheral blood lymphocytes does not necessarily reflect the local immune response at the lung, which is the most clinically important target of SARS-CoV-2; (3) the fact that we did not measure the levels of certain pro-inflammatory cytokines (which are known to be elevated in COVID-19), in an effort to establish a possible link between cytokine levels and lymphocyte numbers. Apart from its prospective design, the strength of our study relies on the fact that the patients were enrolled during the same phase of the disease course, as it is indicated by the similar time points from hospital admission and symptom onset. Moreover, the comparison of the lymphocyte kinetics was made between clinically discrete groups of patients and at separate time points (before and at the end of corticosteroid administration) and thus permitted us to evaluate a possible effect of steroid treatment in these patients.

Overall, we have demonstrated that patients with COVID-19, with or without dexamethasone treatment, exhibited significant differences in blood-lymphocyte kinetics, and these changes were linked to the disease severity and final outcomes. Our findings suggest that certain host characteristics guide immune response in patients with COVID-19 and imply that patients with distinct lymphocytic kinetics during the disease course may differentially benefit from the steroid treatment, a hypothesis that requires further investigation.

## Figures and Tables

**Table 1:** Clinical and epidemiological characteristics of patients on admission.

	No DXM	DXM Success	DXM Failure
Number of patients	42	56	12
Age	53.02 ± 2.138 *^,#^	61.02 ± 1.6	69.58 ± 4.485
Gender Male Female	23 ^#^ 19	24 * 32	11 1
BMI <30 >30	30 12	34 22	10 2
Nationality: European Other	37 5	51 5	11 1
Chest X-ray: <2 quartiles >2 quartiles	38 *^,#^ 4	28 * 28	3 9
CCI	1 (0–2) *^,#^	2 (1–3)	3.5 (1.25–6)
NEWS2 score	2 (1–3) *^,#^	6 (3.25–7)	7 (4.25–8.75)
NEWS2 score: <5 >5	38 *^,#^ 4	17 * 39	3 9
Temperature (°C)	37.89 ± 0.1291	37.96 ± 0.1139	37.83 ± 0.2553
Respiratory Rate (breaths/min)	20 (18–22) *^,#^	25 (20–26)	25.5 (19.75–31.5)
Systolic blood pressure (mmHg)	120 (110–131)	120 (110–130)	130 (112.5–140)
Heart rate (bpm)	89.62 ± 1.96 *	88.82 ± 1.737 *	106.3 ± 6.36

Quantitative variables with normal distribution are presented as mean ± standard error of the mean (SEM). Quantitative variables with non-Gaussian distribution are presented as median (inter-quartile range—IQR). Qualitative variables are presented as numbers. DXM: Dexamethasone. BMI: body mass index. CCI: Charlson Comorbidity Index. NEWS2: National Early Weaning Score 2. *p*-values < 0.05 were considered significant. * *p* < 0.05 to DXM failure, ^#^ *p* < 0.05 to DXM success.

## Data Availability

Data are available upon contact with the corresponding author.

## Supplementary Materials

The following supporting information can be downloaded at: https://www.mdpi.com/article/10.3390/v15010051/s1, Table S1: Laboratory findings of patients on admission, Table S2: Days of samples’ acquisition.

## Abbreviations

DXM: dexamethasone; COVID-19: Coronavirus disease 2019; SARS-CoV-2: Severe acute respiratory syndrome coronavirus 2.
