# Supplementary material for: Kinetics of Immune Subsets in COVID-19 Patients Treated with Corticosteroids
Source: Viruses. 2022 Dec 24;15(1):51. doi: 10.3390/v15010051 (PMC9865280; doi:10.3390/v15010051)
Supplement: Supplementary file 1 [file viruses-15-00051-s001.zip › viruses-2065117-supplementary.pdf]

viruses

MDPI

Table S1. Laboratory findings of patients on admission. Quantitative variables with normal distribution are presented as mean (± sem). Quantitative variables with non-Gaussian distribution are presented as median (inter-quartile range-IQR). Qualitative variables are presented as numbers. DXM: Dexamethasone, WBC: White Blood Cell count, HcT: Hematocrit, HgB: Hemoglobin, PT: Prothrombin Time, aPTT: activated Partial Thromboplastin Time, INR: International Normalized Ratio, ESR: Erythrocyte Sedimentation Rate, AST: Aspartate Transaminase, ALT: Alanine Transaminase, LDH: Lactate Dehydrogenase, CRP: C-reactive protein, CPK: Creatine Phosphokinase. p-values <0.05 were considered significant.$^{*}p<0.05$ to DXM failure, $^{\#}p<0.05$ to DXM success.

	**No DXM**	**DXM success**	**DXM failure**
WBC/μL	4940 (4205–6315)*	5310 (3933–6638)*	7530 (5058–10720)
Platelets X1000/μL	172.5 (144.75–217.75)	189.5 (153–219.75)	148.5 (117.25–232.75)
Hct (%)	39.2 (35.98–41.23)	37.75 (34.78–40.2)	39.75 (36.2–42.5)
HgB g/dL	12.75 ± 0.2928	12.49 ± 0.1921	13.08 ± 0.5845
PT sec	12.45 (11.8–13.13)*	12.7 (12.1–13.2)*	14.1 (12.88–15.68)
aPTT sec	29.9 (27.68–33.25)*, #	32.85 (29.58–37.18)	34.85 (32–46.1)
INR	1.03 (0.975–1.075)*	1.03 (0.9825–1.093)*	1.16 (1.068–1.263)
D-Dimers μg/mL	0.6 (0.47–1.03)	0.72 (0.49–1.06)	0.87 (0.52–1.64)
Ferritin ng/mL	232 (70.1–403.6)	301.7 (156.9–515.2)	382.4 (233.1–968.9)
ESR mm/h	37 (26–46.5)	49 (33–58)	30 (12–56)
Urea mg/dL	29 (22.75–33.5)*	24.5 (20.25–34)*	40.5 (34–61.5)
Creatinine mg/dL	0.8 (0.7–1)*	0.8 (0.7–0.9)*	1.15 (0.9–1.35)
Na^{+} mmol/L	139 (136–141.3)	137 (134–140)	138.5 (137.3–139)
K^{+}mmol/L	4.2 (3.9–4.42)	4.1 (3.8–4.27)	4.15 (3.8–4.47)
AST IU/L	28 (17–42.25)	35 (26–45.75)	33.5 (21.25–78.25)
ALT IU/L	22.5 (16.75–37)	31.5 (18–44.5)	23.5 (19.25–77.75)
Billirubin mg/dL	0.38 (0.2775–0.585)*	0.41 (0.31–0.5175)*	0.76 (0.4525–1.043)
LDH IU/L	245 (190–302.3)*, #	320 (248.3–416.5)	365.5 (247.8–539)
CRP mg/dL	2.25 (1.27–5.37)*, #	7.35 (3.7–12.75)	11.35 (2.8–16.58)
CPK IU/L	89.5 (60.25–162)*	133 (58.75–254)	453 (91–890.8)
Troponine pg/mL	6 (4–10)*	7 (5–12.75)	14.5 (6.75–39.5)
Lactate mmol/L	0.9 (0.7–1)*	0.9 (0.8–1.2)	1.1 (0.9–2.5)

Table S2. Days of samples' acquisition. Values are presented as median (inter-quartile range-IQR). DXM: dexamethasone. *p*-values < 0.001 were considered significant. *: $p<0.001$ to no DXM.

	No DXM	DXM success	DXM failure
**1st Sample**			
Days from admission	2 (1–3)	2 (2–2.75)	1 (1–2)
Days from symptom onset	9 (6–11)	9 (7-11)	9 (6.5–9.75)
**2nd Sample**			
Days from admission	5 (4.5–5.5)	8 (7–10.5) *	11 (9.5–11)*
Days from symptom onset	12 (9–13)	15 (13.5–18)*	17 (16.25–18.75)*
